# Cross-cultural adaptation and validation of the South African Pain Catastrophizing Scale (SA-PCS) among patients with fibromyalgia

**DOI:** 10.1186/1477-7525-10-137

**Published:** 2012-11-22

**Authors:** Linzette D Morris, Karen A Grimmer-Somers, Quinette A Louw, Michael J Sullivan

**Affiliations:** 1 Division of Physiotherapy, Department of Interdisciplinary Health Sciences, Faculty of Medical and Health Sciences, Stellenbosch University, Tygerberg, South Africa; 2 International Centre for Allied Health Evidence (iCAHE), University of South Australia, Adelaide, Australia; 3 Departments of Psychology, Medicine and Neurology, Canada Research Chair in Behavioural Health, McGill University, Montreal, Quebec, Canada

**Keywords:** Fibromyalgia, Chronic pain, Pain catastrophizing, Outcome measures, Pain catastrophizing scale, Cross-cultural adaptation, Validation, Psychometric properties, South Africa

## Abstract

**Background:**

Pain catastrophization has recently been recognized as a barrier to the healthy development of physical functioning among chronic pain patients. Levels of pain catastrophization in chronic pain patients are commonly measured using the Pain Catastrophizing Scale (PCS).

**Objective:**

To cross-culturally adapt and validate the South African PCS (SA-PCS) among English-, Afrikaans- and Xhosa-speaking patients with fibromyalgia living in the Cape Metropole area, Western Cape, South Africa.

**Methods:**

The original PCS was cross-culturally adapted in accordance with international standards to develop an English, Afrikaans and Xhosa version of the SA-PCS using a repeated measures study design. Psychometric testing included face/content validity, internal consistency (Cronbach’s alpha-α), test-retest reliability (intraclass coefficient correlations-ICC), sensitivity-to-change and cross-sectional convergent validity (by comparing the adapted SA-PCS to related constructs).

**Results:**

The cross-culturally adapted English, Afrikaans and Xhosa SA-PCS showed good face and content validity, excellent internal consistency (with Chronbach’s α = 0.98, 0.98 and 0.97 for the English, Afrikaans and Xhosa SA-PCS, as a whole, respectively), excellent test-retest reliability (with ICC’s of 0.90, 0.91 and 0.89 for the English, Afrikaans and Xhosa SA-PCS, respectively); as well as satisfactory sensitivity-to-change (with a minimum detectable change of 8.8, 9.0 and 9.3 for the English, Afrikaans and Xhosa SA-PCS, respectively) and cross-sectional convergent validity (when compared to pain severity as well as South African versions of the Tampa scale for Kinesiophobia and the revised Fibromyalgia Impact Questionnaire).

**Conclusion:**

The SA-PCS can therefore be recommended as simple, efficient, valid and reliable tool which shows satisfactory sensitivity-to-change and cross-sectional convergent validity, for use among English, Afrikaans and Xhosa-speaking patients with fibromyalgia attending the public health sector in the Western Cape area of South Africa.

## Background

Over the past few decades, the role of pain catastrophization in the development and maintenance of chronic pain has gained considerable attention in research and clinical practice [1-6]. A cognitive strategy broadly defined as “an exaggerated negative orientation towards actual or anticipated pain experiences”; pain catastrophization has shown associations with functional disability, pain severity, elevated disease activity and depression in chronic pain patients [6-8]. Assumed to be more pronounced in patients with fibromyalgia than in any other chronic pain population; pain catastrophization often leads to the development of fear-avoidance behaviours and non-adherence towards prescribed exercise programs among patients with fibromyalgia [5,9,10]. Since inactivity among patients with fibromyalgia is particularly detrimental and typically results in deconditioning of the musculoskeletal system and increased fibromyalgia symptoms; the presence of pain catastrophization poses various challenges to the effective management and maintenance of physical function among patients with fibromyalgia [8,10,11]. Pain catastrophization is thus currently recognized as a barrier to the healthy development of psychological and physical functioning among patients with fibromyalgia [6,12].

One of the most common outcome measures used to subjectively quantify pain catastrophization in clinical practice and research is the Pain Catastrophizing Scale (PCS) [13,14]. Initially developed in English by Sullivan et al. in 1995; the original PCS is considered a ‘broader, reliable and valid measure of catastrophization” [7]. The items integrated into the original scale were specifically intended to assess elements of pain catastrophization [7,14]. The PCS is thus a useful tool to identify individuals who may be susceptible to amplified negative responses towards pain and the anticipation of pain [7]. Numerous validation studies have since shown that the PCS has a concrete factor structure, credible psychometric properties (i.e. internal consistency, test-retest reliability and validity) and is correlated to other health outcomes such as pain intensity, pain-related disability, fear-avoidance behaviours and psychological distress [15,16].

Similar to most developing worlds, chronic pain epidemiologic information for South Africa was until recently very limited [17-19]. For many years, research was largely prioritized to epidemics such as HIV/Aids and tuberculosis [20]. The recent postulation that chronic pain conditions may be more prevalent in developing nations and in fact place a higher burden on constrained health budgets, has shifted the focus in research [18,19,21,22]. Reflecting the global burden of chronic pain conditions; there is a growing recognition of the importance of accurate outcome measurement and comparability of outcomes across developed and developing world chronic pain populations [23,24]. However, populations and cultural sub-groups within populations across the globe, typically differ in language, dialect, lifestyle, morals, values, behaviour, customs, beliefs, perceptions of life and expression of disease [25-27]. The direct administration of existing and previously validated versions of self-reported outcome measures in various countries, cultures and language groups, is therefore not always possible or advised. In these instances, misinterpretations of the questions or scoring systems, and culturally-inappropriate anchores or references, may compromise the integrity and validity of the responses that the outcome measure seeks [25,27-29]. The ramifications of utilizing linguistically- or culturally-inappropriate health outcome measures across various populations and cultures are therefore far-reaching; not only in terms of decisions made on effective care, but also in terms of health policies which may be developed from the research findings. Accurate measurement across cultures is thus dependent on the proper linguistic and cultural adaptation and application of an outcome measure for a specific population [27-30].

In other countries, the cross-cultural adaptation and validation of an outcome measure is typically reported for one cultural/ethnic group in one predominant language. However, in a country like South Africa where vast diversity exist with regards to language, ethnicity, socioeconomics, culture and religion; the implementation of an outcome measure in one language is not possible. A total of 11 official languages are spoken throughout the country, with the dominance of each language varying between different parts of South Africa [31]. Afrikaans is arguably the most predominant language spoken in the western parts of South Africa, with 55.3% of the Western Cape population speaking Afrikaans as a first language. Xhosa is the first language of 23.7% and English, the first language of 19.3% of the population. The Western Cape population comprises of 50.2% “coloured”; 30.1% “blacks”; 18.4% “whites” and 1.3% “India/Asian or other” [32]. In the “coloured” community, 81% speak Afrikaans as a first language; and in the “black” community 88.6% speak Xhosa as a first language. The “white” community is however evenly divided between Afrikaans- and English-speaking, at 55.4% and 43.2%, respectively [32]. Conversely, in the northern parts of South Africa, “blacks’ are the predominant ethnic group, and the majority of this group speak Zulu. In the eastern parts, the predominant ethnic groups are “Indian” and “blacks”, and the main languages are English and Zulu. The implementation of outcome measures cross-culturally adapted, translated and validated in only one language or ethnic group is therefore inappropriate among the South African population where vast diversity exists.

The MAPI institute is an international company which currently offers services relating to linguistic validation of patient-reported outcome measures [33]. With more than 20 in-house translators, the institute is dedicated to producing accurately translated versions of most patient-reported outcome measures for use cross-culturally in health research across the globe. Currently, 18 translated versions of the PCS are registered on the MAPI database, including an Afrikaans version which was specifically translated for use within a South African context. This Afrikaans version of the PCS has however, to date not been validated among a South African fibromyalgia population. The following 11 translated versions available on the MAPI database have subsequently been cross-culturally adapted and validated among various healthy and chronic pain populations, including patients with fibromyalgia, namely; the Italian-, Croatian-, Singhalese-, Spanish-, German-, Catalan-, Chinese-, French-, French-Canadian-, and Dutch-PCS [14,34-42]. Since the Afrikaans version of the PCS available from the MAPI institute database has yet to be validated among a South African population, and no Xhosa version of the PCS currently exists; further adaptation and validation of the PCS is required to ensure cultural and linguistic applicability within a South African fibromyalgia population. The purpose of the following study was thus to cross-culturally adapt and validate the South African PCS (SA-PCS) for use among English-, Afrikaans- and Xhosa–speaking patients with fibromyalgia living in Western Cape area of SA.

## Methods

Ethical approval for this study was obtained from the Health Research Ethics Committee of the Stellenbosch University, South Africa, during July 2010. Permission to conduct the study at the Tygerberg Hospital an academic tertiary institution situated in the northern suburbs of the Cape Metropole area of the Western Cape, South Africa was granted by the Western Cape Department of Health. The institution is dedicated to providing healthcare services to people living in and around the Cape Metropole area, as well as people from the broader Western Cape area. Permission to cross-culturally adapt and validate the original English and existing Afrikaans version of the PCS, and to translate the adapted versions into Xhosa was obtained from the original developer of the PCS, Prof. Michael J. Sullivan. All eligible subjects were required to read and sign an informed consent form prior to participating in the study. To ensure anonymity, a unique study identification number/code was allocated to each subject on recruitment. Confidentiality of subject information and data was maintained by storing all study data in a locked, access-controlled facility.

This study incorporated a repeated-measures study design.

### Subjects

Subject recruitment criteria included:

Male and female adults aged 18 years and older;

Patients clinically diagnosed with fibromyalgia according to the American College Rheumatology (ACR) criteria by a qualified rheumatologist;

Patients with fibromyalgia registered at the Tygerberg Hospital’s Rheumatology clinic;

South African citizens;

Patients with fibromyalgia who spoke, comprehended and were proficient in either the English, Afrikaans or Xhosa language;

Patients with fibromyalgia who resided in and around the Cape Metropole area or the larger part of the Western Cape area of South Africa

Subjects who were not fully comprehensive of what the project entailed and what was expected of them, and for whom valid contact details were unavailable from the rheumatology clinic’s database, were excluded. Eligible subjects were consecutively recruited into the study.

### Sample size calculation

The main objective of this study was to ascertain the validity of the cross-culturally adapted English, Afrikaans and Xhosa versions of the PCS. For internal consistency, the sample size required for this study was based on an intraclass coefficient correlation (ICC) of 0.9 and a maximum width of 0.23 for the 95% confidence interval (CI). The expected ICC and width of the 95% CI was based on previous studies. The formula used to calculate the sample size was N = [16p (1-p)]/w^2^ where *p* is the expected ICC (0.9) and *w* is the width of the 95% CI. The minimum total sample size per language group was therefore calculated to be 27 subjects.

### Study instruments

The original English PCS

A self-report measure, the PCS is a broad measure of pain catastrophizing and consists of 13 items scored using a 5-point Likert scales from 0 (never) to 4 (always) points. The total score for the PCS equals 52. Responses are summed to create a total score, with higher scores indicating greater pain catastrophizing levels. A score of more than 24 indicates a high level of catastrophizing. The items are divided into three subscales; namely *rumination, helplessness* and *magnification*. Rumination (items 8–11) “refers to the fact that the patient cannot get the idea of pain out of his/her head and cannot stop thinking about the pain”; Helplessness (items 1–5 and 12) “refers to the estimation that the person has of not being able to do anything to influence the pain”; and Magnification (items 6, 7 and 13) “refers to the exaggeration of the threatening properties of the painful stimulus”. High internal reliability (Chronbach’s alpha (α) for total PCS = 0.9) has been reported in patients with chronic pain with adequate validity and test-retest reliability [7].

Pain severity scale

Pain severity was measured using a simple 5-point Likert scale where “1”was “not bad at all” and “5” was ”unbearable”.

Tampa scale for Kinesiophobia (TSK)

The TSK is a self-report instrument designed to assess fear of pain and activity. It consists of 17 items each rated on a 4-point Likert scale. The total score for the TSK equals 68. Responses are summed to create a total score, with higher scores indicating greater fear of pain and activity. A score of more than 37 on the TSK indicates a high level of fear. The scale has demonstrated test-retest reliability and internal consistency (Cronbach alphas have ranged 0.7 to 0.8) in studies of patients with chronic low back pain. Stability over time and the criterion validity and construct validity have been well established [43].

Revised Fibromyalgia Impact Questionnaire (FIQR)

The FIQR is an updated version of the Fibromyalgia Impact Questionnaire (FIQ) that has good psychometric properties, can be completed in less than two minutes and is easy to score. It has scoring characteristics comparable to the original FIQ, making it possible to compare past FIQR results with future FIQR results [44]. The original FIQ was developed and validated by Burckhardt et al. (1991) to assess the current health status of women with fibromyalgia [45].

General Practice Physical Activity questionnaire (GPPAQ)

The GPPAQ is a validated screening tool for use in primary care to assess adult (16 – 74 years) physical activity levels [46]. It provides a simple 4-level physical activity index, categorizing patients as inactive, moderately inactive, moderately active and active.

Sociodemographic form

The form was specifically designed to collect data pertaining sociodemographic information (i.e. age, gender, marital status, level of education, employment status, etc.) as well as information pertaining to fibromyalgia pain/symptoms, severity of pain /symptoms, frequency of pain/symptoms, etc.).

### Study procedure

Eligible subjects were sampled from the available fibromyalgia population registered at the Tygerberg Hospital’s Rheumatology clinic, and those attending the fibromyalgia support group (hosted by the Tygerberg Hospital’s Occupational Therapy department) during October 2010 and December 2011. The rheumatologists and/or occupational therapist working at the clinic were requested to identify all new or previously diagnosed patients with fibromyalgia. In addition, the principal researcher and research assistant manually searched the clinic’s database for any patients with fibromyalgia discharged from the clinic between 2009 and 2011. Patients were identified using the current ICD-10 code for fibromyalgia, viz. M79.9. All patients for whom contact details were available and valid, were contacted telephonically and invited to participate in this study. A professional Xhosa translator assisted in collecting data from Xhosa- speaking participants. On recruitment into the study, the study procedure was thoroughly explained to each eligible subject and an informed consent form was read and signed in their preferred language. Information pertaining to sociodemographics; fibromyalgia pain/symptom severity and frequency; and general physical activity level was collected for all eligible subjects on recruitment using specifically designed forms. Remuneration for travelling costs to and from the study setting was provided.

### Cross-cultural adaptation process

Cross-cultural adaptation of the original English and Afrikaans versions of the PCS (provided by the MAPI institute) was performed in accordance with previously published guidelines [24,26]. Two rheumatologists, one pain physiotherapist and two occupational therapists, knowledgeable in the field of fibromyalgia/rheumatology and working in the public/private sector were invited to form part of an expert committee and assist in the cross-cultural adaptation of the PCS. A subgroup of ten patients (five English- and five Afrikaans-speaking) with fibromyalgia currently attending the fibromyalgia support group hosted on a monthly basis by the Tygerberg Hospital’s Occupational therapy department, were invited to participate in this preliminary process. The original English and Afrikaans versions of the PCS were sent to the panel members via email, and were personally administered to the subgroup of subjects with fibromyalgia in a scheduled meeting. In a detailed letter, the panel members and subjects were requested to carefully check if there were any items in the original English and Afrikaans versions of the PCS which were not applicable to patients with fibromyalgia currently registered at the Tygerberg Hospital’s Rheumatology clinic and living in South Africa. The panel members and subjects with fibromyalgia were further requested to suggest any changes which may deem the questions more applicable to the intended population. After the PCS versions had been reviewed and scrutinised by the panel members and the subgroup of subjects with fibromyalgia, the principal researcher collated the suggested changes in MS Excel.

Based on the suggestions and comments received by the expert committee and the subgroup of patients with fibromyalgia, changes were made to the overall layout, the instructions, the scoring system, and wording of the items of the original versions of the PCS, to make it more culturally applicable for South African patients with fibromyalgia. The instructions on how to complete the PCS were revised and simplified, and the section pertaining to the statement regarding individual pain experiences was removed. For the purpose of this validation study, the section pertaining to personal information was removed and replaced with a section for the study identification number and the date.

The anchores “not at all”, “to a slight degree”, “to a moderate d*egree”, “to a great degree”* and *“all the time”* were retained in the adapted version of the PCS. However, since patients found it difficult to understand and apply the original scoring system, modifications were made to the layout of the form by placing tick boxes next to each question individually for the anchores. Patients were therefore required to place their responses using an ‘X’ in the appropriate box. The score for each anchore remained the same as the original PCS, ranging from “0” for “not at all” to “4” for “all the time”.

A number of changes were made to the wording of the items based on the suggestions made by the expert committee and the patient group. In the original PCS, the words “*When I’m in pain…*” is placed at the top of the item list and is required to be applied to each item individually. Patients in this sample, however, had difficulty understanding this concept. A modification to the original PCS was therefore implemented whereby the words “*When I’m in pain*…*”* was placed before each item individually.

### Forward- and back translation process

A professional and independent freelance Afrikaans and Xhosa translator and a native Afrikaans- and Xhosa speaking health professional were consulted for English to Afrikaans and Xhosa forward-translation of the adapted English SA-PCS to ensure that all changes suggested during the cross-cultural adaptation process were incorporated. The translators were not informed of the project details. The translators were asked to compare the translated Afrikaans and Xhosa versions of the SA-PCS to the original PCS and the adapted English SA-PCS. The translated Afrikaans and Xhosa versions of the SA-PCS were forwarded to two professional Afrikaans and Xhosa translators who independently and blindly performed back translations of the Afrikaans and Xhosa SA-PCS into English. The English SA-PCS was not supplied to the back translators as a reference. Following professional forward- and back-translation of the English SA-PCS to Afrikaans and Xhosa, as well as professional editing of all the documents, the face/content validity of the pre-final versions of the English, Afrikaans and Xhosa SA-PCS was tested.

#### Psychometric and statistical methods

Data were collated, extracted and entered into a purpose-built MS Excel worksheet. Incomplete or incorrectly completed SA-PCS forms were considered and analyzed accordingly. Descriptive statistics were used to analyse data collected pertaining to socio-demographic information, fibromyalgia symptoms and general physical activity levels.

### Face and content validation

The pre-final version of the English, Afrikaans and Xhosa SA- PCS were tested among a second subgroup of subjects with fibromyalgia to establish face and content validity (acceptability and comprehensibility). The subjects were given the SA-PCS in their preferred language (English, Afrikaans or Xhosa). The purpose of this procedure was to ensure that the cross-culturally adapted and translated versions of the SA-PCS were understood within the local context and in the provided languages, and that the items measured what they were intended to measure [47]. The subjects had the opportunity to scrutinize the content of the cross-culturally adapted and translated versions of the SA-PCS and comment on the ease of completing the questions. The subjects were asked questions based on their understanding of the instructions provided; the ease of understanding the questionnaire in their language; the ease of completing the questionnaire; and if sufficient time was provided to complete the questionnaire. Any suggestions as to how the items could be improved to ensure clarity were documented and considered, and changes were made accordingly. The time taken to complete the pre-final version of the SA-PCS was recorded for each subject. Open-ended responses collected during the face and content validity testing were coded and qualitatively analyzed. Based on the subjects’ responses, comment and suggestions; the necessary changes were incorporated and the final versions of the English, Afrikaans and Xhosa SA-PCS were produced for further testing of the following psychometric properties, namely: *internal consistency, test-retest reliability, sensitivity-to-change* and *cross-sectional convergent validity*.

### Internal consistency

Internal consistency relates to the homogeneity of the scale and how well the items on a tool fit together conceptually [48]. Internal consistency of the final cross-culturally adapted and translated SA-PCS was estimated using the Cronbach’s alpha (α) that ranges from 0–1. Chronbach’s α indicates the strength of the relationship between all the items with the measurement tool [47,49]. Siegle’s (2005) Reliability Calculator, an MS Excel add-on, was used to calculate the Chronbach’s α estimates for the subsections of the SA-PCS (rumination, helplessness and magnification) and the total SA-PCS [50]. The internal consistency was estimated for each version of the SA-PCS separately; the English, Afrikaans and Xhosa SA-PCS as a whole.

### Test-retest reliability

Test-retest reliability measures stability over time/reproducibility and is relevant for cognitive and trait scales which are not expected to change over time [48]. Subjects were required to complete two SA-PCS forms at different time points, one month apart. To evaluate test-retest reliability, intraclass correlation coefficient (ICC) and 95% confidence intervals (CIs), as well as standard error of measurement (SEM) were estimated. The ICC is “an index of the reliability of the measurements between tests” [51,52]. One-way ANOVA’s were conducted to calculate the within-subject/between-subject variance which was used to calculate the ICC. ICC values of 0.6 to 0.8 were regarded as evidence of good reliability, and those of higher than 0.8 were considered as excellent reliability [18]. The higher the coefficient value, the higher the reliability and the lower the standard error of measurement. The SEM “estimates how repeated measures on the same instrument tend to be distributed around the “true” score [53].

### Sensitivity-to-change

Sensitivity to change is defined as “the capacity of a measure to detect change in patients over time” [54] and relates to the “clinical meaningfulness of changes in scores” [55]. Sensitivity to change was estimated using the minimum detectable change (MDC) which was calculated by multiplying the SEM by the z-score associated with the 95% confidence interval and the square root of 2, which reflects the additional uncertainty introduced by using difference scores based on the measurements made at two time points (one month apart) [34]. The MDC is “the degree of change required in an individual’s score to ascertain if the change is real, over and above measurement error” [34].

### Cross-sectional convergent validity

*Cross-sectional convergent validity* is defined as “the extent to which the scores of the measurement of interest relate to other measures in an expected manner” [48]. Usually, the adapted measure is correlated with a related measure which has been previously validated in a similar population or ‘gold standard’. However, since no ‘gold standard’ currently exists for pain catastrophization and no related outcome measure has been previously validated in a South African fibromyalgia population; the scores of the adapted PCS was correlated to scores obtained from the adapted and translated South African versions of the TSK and FIQR, as well as pain severity. Pearson correlation coefficients (*r*) were used to measure *cross-sectional convergent validity* between the adapted and translated PCS and the TSK and FIQR. Student’s t-tests were used to estimate the significance of correlations. Statistical significance was accepted at *p* < 0.05. It was hypothesized that the adapted and translated English, Afrikaans and Xhosa versions of the PCS would measure pain catastrophization scores relative (in a positive direction) to SA-TSK and SA-FIQR scores, as well as pain severity.

## Results

**Figure 1:**
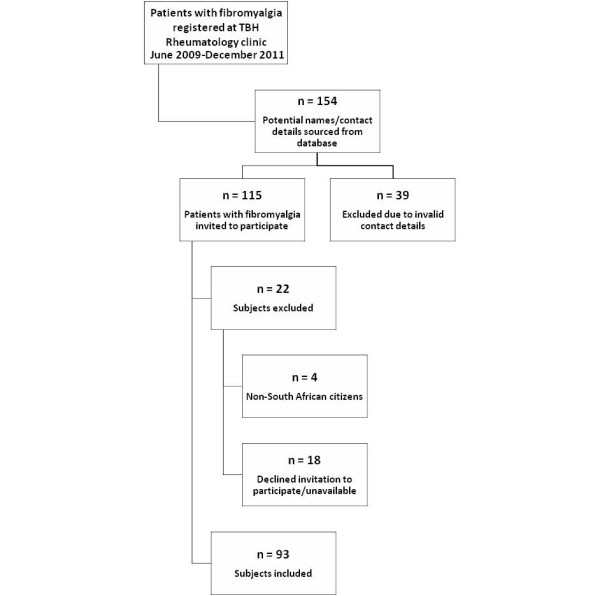
Flow diagram depicting subject inclusion and exclusion process.

A total of 154 patients diagnosed with fibromyalgia between June 2009 and December 2011, were identified either by the rheumatologists working at the Tygerberg Hospital’s Rheumatology clinic, the Tygerberg Hospital’s Occupational Therapy department or via a search of the clinic’s database. Figure 1 illustrates the inclusion and exclusion process of subjects.

### Subject characteristics

**Figure 2:**
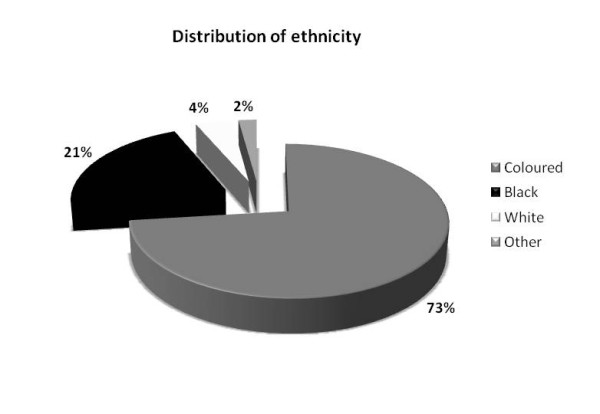
Distribution of ethnicity per language group.

Ninety-three eligible subjects with fibromyalgia (89 females and 4 males) were included in this study. The mean (±SD) age for the subjects was 47.3 (±10.4) years. The mean (±SD) number of years living with fibromyalgia was 5.4 (±4.9) years. The ethnic groups included “coloured” (n=68; 73.1%); “black” (n=19; 20.4%); “white” (n=4; 4.3%) and “other” (n=2; 2.2%). Among this group, 41 were Afrikaans-speaking, 33 were English-speaking and 19 were Xhosa-speaking. Figure 2 depicts the distribution of ethnic group per language.

Thirty-four (36.6%) subjects were married and the mean (±SD) number of children was 3 (±1). The highest level of education for the majority of the subjects (n=38; 40.9%) was reported as lower than grade 12. Table 1 illustrates the distribution of languages and ethnic group for each educational level category: “lower than grade 7; grade 7; lower than grade 12; grade 12; and tertiary education”.

**Table 1:** Distribution of ethnic group and language per education level

	**LEVEL OF EDUCATION**					
**Language/ ethnic group**	**lower than Grade7**	**Grade7**	**lower than Grade 12**	**Grade 12**	**Tertiary education**	**Total**
**Afrikaans**	**3**	**1**	**21**	**14**	**2**	**41**
Coloured	3	1	20	14	2	40
White			1			1
**English**	**3**	**5**	**16**	**5**	**4**	**33**
Coloured	2	5	13	5	3	28
Other			2			2
White	1		1		1	3
**Xhosa**		**12**	**1**	**6**		**19**
Black		12	1	6		19
**Total**	**6**	**18**	**38**	**25**	**6**	**93**

**Figure 3:**
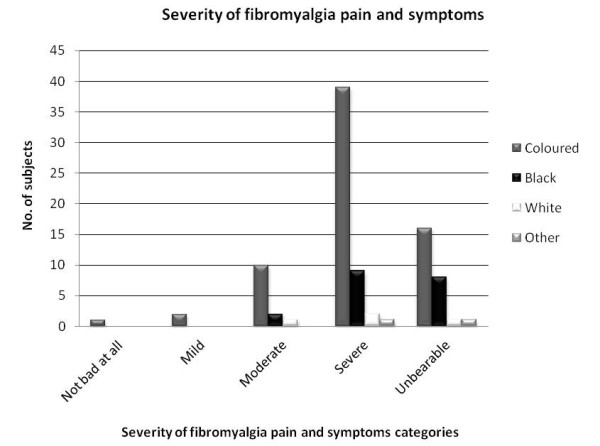
Distribution of severity of fibromyalgia pain and symptoms.

The majority of subjects reported to be unemployed (n=32; 34.4%). Of those reported as “unemployed”, 17 (53.1%) were “coloured” and 14 (43.8%) were “black”. The mean (±SD) number of years living with fibromyalgia was 5.2 (5.5). Severity (measured as “not at all”; “mild”; “moderate”; “severe” and “unbearable”) and frequency (measured as “all the time”; “everyday”; “every second day”; “2 to 3 times per week” and “once a week”) of pain and symptoms related to fibromyalgia were reported. Figure 3 illustrates the distribution of severity of fibromyalgia pain/symptoms among the included subjects.

### Face and content validation of the SA-PCS

A subgroup of 24 subjects (eight English-; nine Afrikaans-; and seven Xhosa-speaking) were invited to test the pre-final versions of the adapted English, Afrikaans and Xhosa SA-PCS. The comments and suggestions made by the patients were collated and evaluated. The mean (±SD) time to complete the English, Afrikaans and Xhosa SA-PCS was 4.8 (±1.2) minutes. All the English-, Afrikaans- and Xhosa-speaking patients with fibromyalgia reported that they understood the instructions provided; that the questionnaire was simple to complete; that the questionnaire was easy to understand in their language; that they understood what was meant by each question and that they were given enough time to complete the questionnaire. No further changes to the adapted English, Afrikaans and Xhosa versions of the PCS were therefore required and the final versions were produced and further validated.

### Internal consistency

Internal consistency was conducted among 93 subjects with fibromyalgia. There were no missing items. Internal consistency of the adapted English, Afrikaans and Xhosa SA-PCS was evaluated by calculating the Chronbach’s α for the subsections (rumination, helplessness, magnification) and the total SA-PCS, which are depicted in Table 2.

**Table 2:** Chronbach’s α values for English, Afrikaans and Xhosa SA-PCS (subsections and totals)

**SA-PCS**	**N**	**Ruminationα**	**Helplessnessα**	**Magnificationα**	**Total SA-PCSα**
Afrikaans SA-PCS	41	0.99	0.98	0.99	0.98
English SA-PCS	33	0.98	0.98	0.96	0.98
Xhosa SA-PCS	19	0.98	0.95	0.93	0.97

### Test-retest reliability

Sixty-seven subjects (26 Afrikaans-; 22 English- and 19 Xhosa-speaking) completed two sets of the SA-PCS in their preferred language, one month apart. Therefore, test-retest reliability analysis was conducted on only 67 of the total 93 subjects. There were no missing items. The ICC’s (95% CIs) and mean differences (MD) were calculated to establish test-retest reliability for the subsections (rumination, helplessness and magnification) and total English, Afrikaans and Xhosa SA-PCS and are depicted in Table 3. No significant differences (*p > 0.05)* were found between the test-retest total and subsection scores of the English, Afrikaans and Xhosa SA-PCS.

**Table 3:** Estimates for test-retest reliability (ICC) of the SA-PCS

**SA-PCS**	**N**	**Max score**	**PCS Test Mean (SD)**	**PCS Retest Mean (SD)**	MD *t2-t1	**ICC (95% CIs)**	**SEM**	**MDC**	***p***
**Afrikaans SA-PCS**	26	**52**	**37.0 (11.4)**	**36.8 (11.3)**	**−0.23**	**0.91 (0.81-0.96)**	**3.3**	**9.0**	**0.71**
Rumination		16	10.2 (3.6)	10.4 (3.6)	0.15	0.88 (0.75-0.94)	1.7	4.7	0.24
Helplessness		24	17.9 (5.0)	17.9 (4.9)	0.01	0.87 (0.74-0.94)	1.8	4.9	0.36
Magnification		12	8.2 (2.9)	8.4 (2.9)	**0.15**	0.86 (0.72-0.94)	1.1	2.9	0.37
**English SA-PCS**	22	**52**	**38.2 (11.5)**	**39.1 (11.5)**	**0.82**	**0.90 (0.78-0.96)**	**3.2**	**8.8**	**0.93**
Rumination		16	10.34 (3.5)	10.4 (3.6)	0.07	0.88 (0.71-0.94)	1.2	3.3	0.77
Helplessness		24	18.2 (4.9)	18.1 (4.7)	−0.10	0.87 (0.79-0.92)	1.7	4.8	0.68
Magnification		12	8.4 (2.7)	8.5 (2.7)	0.09	0.86 (0.70-0.94)	1.0	2.8	0.57
**Xhosa SA-PCS**	19	**52**	**34.2 (8.5)**	**32.9 (8.8)**	**−1.27**	**0.89 (0.74-0.96)**	**3.3**	**9.3**	**0.79**
Rumination		16	10.5 (3.5)	10.7 (3.5)	0.20	0.88 (0.71-0.95)	1.2	3.4	0.37
Helplessness		24	18.2 (4.8)	18.1 (4.6)	−0.15	0.86 (0.67-0.94)	1.7	4.9	0.59
Magnification		12	8.4 (2.7)	8.6 (2.6)	0.11	0.84 (0.63-0.93)	1.0	2.9	0.52

*t2-t1 = mean difference between test and retest scores; ICC = intraclass correlation coefficient; SEM = standard error of measurement;

MDC = minimum change difference; MD = mean difference; 95% CIs = 95% confidence intervals.

### Sensitivity-to-change

The MDC were calculated for the SA-PCS as a whole and for the subsections, to establish the smallest change needed in scores to reflect a true change rather than measurement error (see Table 3). The MDC for the English, Afrikaans and Xhosa SA-PCS, as a whole, was 8.8, 9.0 and 9.3, respectively.

### Cross-sectional convergent validity

The scores of the English, Afrikaans and Xhosa SA-PCS for the total sample (n=93) were correlated to the scores of the following related outcome measures: *pain severity (5-point Likert scale), fear-avoidance behaviour (TSK) and impact of fibromyalgia (FIQR)*. The Pearson’s correlation coefficients (*r*) produced between the various outcome measures are depicted in Table 4. Significant correlations were found between the English (*p* = 0.011), Afrikaans (*p* = 0.004) and Xhosa SA-PCS (*p* = 0.038) and the TSK; and between the Afrikaans SA-PCS and the FIQR (*p* = 0.049).

**Table 4:** Pearson’s correlations between various outcome measures

**SA-PCS**	**N**	**Pain severity*r***	***p***	**SA-TSK*r***	***p***	**SA-FIQR*r***	***p***
Afrikaans SA-PCS	41	0.25	0.228	0.55	0.004†	0.39	0.049†
English SA-PCS	33	0.32	0.147	0.53	0.011†	0.38	0.081
Xhosa SA-PCS	19	0.30	0.212	0.48	0.038†	0.30	0.212

†*Significance set at p<0.05.*

SA-TSK = South African Tampa scale for Kinesiophobia; SA-FIQR = South African revised Fibromyalgia Impact Questionnaire.

## Discussion

The aim of this study was to cross-culturally adapt and validate the PCS for use in English-, Afrikaans- and Xhosa-speaking patients with fibromyalgia living in the western parts of South Africa. Modifications to the wording of the items and scoring system were required to ensure that the PCS would be applicable within a South African context. The study results show that the English, Afrikaans and Xhosa SA-PCS are valid and reliable tools for administration within the public health sector among South African patients with fibromyalgia living in the Western Cape (South Africa).

As anticipated, cross-cultural adaptation and validation of the PCS for the South African context was complex because of the cultural and linguistic variability evident in various areas of this diverse country. Although the SA-PCS was cross-culturally adapted and validated in three of the most predominant languages of the western part of South Africa, the translated and validated instruments resulting from this study may not be applicable to Zulu- or Sesotho-speaking patients with fibromyalgia living in the northern and eastern parts of South Africa. The instrument adaptations from this study may also not be applicable for, or accepted by, other English-, Afrikaans- and Xhosa-speaking ethnic and culture groups living in the other parts of South Africa. Careful consideration for diversity is therefore required when applying any health outcome measure among various languages, cultural or ethnic groups uniquely found in South Africa. Further cross-cultural adaptation and validation of the SA-PCS in other South African language and ethnic groups is therefore recommended.

Face and content validation identified that the English, Afrikaans and Xhosa SA-PCS were acceptable, applicable and easily comprehended by the included subjects. The English, Afrikaans and Xhosa SA-PCS were also simple to complete as it took subjects less than five minutes to complete the questionnaire. Furthermore, the adaptation and application of the scoring system proved to be easier for the subjects to understand what was expected. We anticipated that complicated scoring systems for outcome measures would be ineffective if the target group does not fully understand how the system works, and how the system should be applied. As a result, incorrect responses and inaccurate study results and conclusions may be obtained. In South Africa, clinicians working in the public health sector particularly have limited time to consult with individual patients due to limited resources and staff [56]. The time taken and the ease of completing a questionnaire, in addition to the cultural applicability of an outcome measure, therefore need to be considered. Accurate measurement is essential before, after and during all management programs for determining the progress of management and the effectiveness of a treatment. It is as important to ensure that acquiring these measures from patients is not frustrating for the health professional and the patient and that clinicians do not neglect assessing outcomes on a regular basis due to time constraints [56]. The English, Afrikaans and Xhosa SA-PCS are therefore simple, efficient, easy to understand, easy to complete and valid tools to use among South African patients with fibromyalgia receiving services the public health sector in the Western Cape (South Africa). Further validation is however required for application of the English, Afrikaans and Xhosa SA-PCS in the South African private sector and in research studies.

Internal consistency for the English, Afrikaans and Xhosa SA-PCS, as a whole, was excellent (α = 0.98, 0.98 and 0.97 respectively). These estimates are higher than the original English PCS (α = 0.87) as well as the Spanish PCS (α = 0.79), Dutch PCS (α = 0.85), French PCS (α = 0.85); Singalese PCS (α = 0.89), Catalan PCS (α = 0.89); Italian (α = 0.92), Chinese PCS (α = 0.93) and German PCS (α = 0.94) [7,34-42]. However, the evaluation of internal consistency of the entire PCS is theoretically incorrect since, by definition, Cronbach’s alpha “indicates the correlation among items that measure one single construct” [49]. The PCS contains three dimensions; hence evaluation of the internal consistency of each of the three subsections is required. The internal consistency for all subsections (rumination, helplessness and magnification) of the SA-PCS was also found to be excellent and considerably higher than previously reported ICC’s for subsections of the PCS. Nevertheless, the high internal consistency for the subsection magnification of the English, Afrikaans and Xhosa SA-PCS (α = 0.96; 0.99 and 0.93 respectively) found in this study is contradictory to the majority of validation studies which have previously reported that internal consistency for the magnification subsection, in particular, is usually unsatisfactory [7,34,37,38,40,41]. The internal consistency reported for the subsection magnification of the original, French, French-Canadian, Catalan, Italian and German PCS ranged between α = 0.56 to 0.67. It has been postulated that the low internal consistency found for the subsection magnification may relate to the few items contained to this subsection and that it should be reconsidered if this subsection can be reliably used as an independent instrument [49]. The higher internal consistency reported in this study may be due to the fact that subjects took more time to answer each question and may have considered each question more carefully, increasing the internal consistency for this subsection. The Chinese PCS reported an internal consistency of α = 0.77, which was closest to that of this study [39]. Further validation of the psychometric properties of the English, Afrikaans and Xhosa SA-PCS among larger sample groups is however warranted.

The SA-PCS showed excellent stability (test-retest reliability) as a whole with no significant difference between test and retest scores, for one month correlation: English SA-PCS (ICC = 0.90), Afrikaans SA-PCS (ICC = 0.91) and Xhosa SA-PCS (ICC = 0.89) (Table 4). These results were higher than the original English (ICC = 0.73), French (ICC = 0.73) and the Catalan versions (ICC = 0.76); were comparable to the Spanish (ICC = 0.84), German (ICC = 0.83), Italian (ICC = 0.84) and French-Canadian (ICC = 0.85) versions; but were lower than those reported for the Dutch (ICC = 0.92) and the Chinese (ICC = 0.96) versions of the PCS [7,34,36-42]. Excellent stability was also found for the subsections (rumination, helplessness and magnification) of each version of the SA-PCS with no significant differences between test and retest scores, which is analogous with previously reported ICCs for the subsections of the PCS [7,34,36-42]. The ICC values obtained for an outcome measure is however largely dependent on variance of disease patterns between subjects and it is acknowledged that the time period between the test and retest influences the size of this variance [48,57]. The longer the time period between the test and retest, the more likely variance between subjects may occur and the lower the ICC value. Conversely, if the period between the test and retest is too short, there is a possibility that recall bias may occur, resulting in a higher test-retest correlation [48]. In chronic pain studies, there is also a good possibility that the results obtained will differ between individuals who are experiencing pain or symptoms at the time of the testing and those who are symptom-free [57]. According to Lamé et al. (2008), the latter group often responds to questions by trying to remember how they feel when they are actually experiencing pain or symptoms than what they are feeling at the time of inquiry [57]. However, due to the chronicity of fibromyalgia, rapid changes in general health, pain/symptom patterns and disability are usually not expected, and the timing and experiences of pain and symptoms naturally vary. The period between the test and retest should therefore be based on the usual clinical practice of most outpatient public health facilities in South Africa where patients often have to wait a few weeks to months between treatments [48,57]. Since the included subjects with fibromyalgia were believed to not vary significantly in general health, pain/symptom patterns or disability within a short time period, the one month period used between the test and retest was deemed appropriate to ascertain reproducibility of the English, Afrikaans and Xhosa SA-PCS.

Sensitivity-to-change was also satisfactorily demonstrated in the English, Afrikaans and Xhosa SA-PCS. At a 95% confidence level, the MDC of the English, Afrikaans and Xhosa SA-PCS, as a whole, indicates that a change of more than 8.8, 9.0 and 9.3 points after a given intervention, respectively, would not be due to measurement error. These values are slightly lower than those reported for the Italian (10.5) and German PCS (12.8) [34,37].

It is acknowledged that a major limitation to this study was that the SA-PCS should have been correlated with a ‘gold standard’. However, since no such measure currently exists for pain catastrophization, the PCS is usually correlated with related outcome measures such as intensity of pain/symptoms, disability, fear-avoidance behaviours or depression, to establish cross-sectional convergent validity [8]. However, a number of related outcome measures had to be cross-culturally validated prior to their use within the South African context. The cross-cultural adaptation and validation of the PCS, TSK and FIQR for a South African fibromyalgia population was therefore concurrently conducted. It was therefore decided that for this study it would be appropriate to correlate the scores of the English, Afrikaans and Xhosa SA-PCS to the English, Afrikaans and Xhosa versions of the TSK and FIQR. Nevertheless, criticism may be justified as to why the SA-PCS was not correlated with intensity of pain/symptoms, such as the Numerical Pain Rating Scale or the Visual Analogue Scale which are widely-accepted valid outcome measures. Instead, in the current study severity of pain/symptoms was measured and subjects were required to report on the everyday activities which increased their pain and symptoms. To defend our research approach, at the time of conceptualizing the study, it was understood that in chronic pain patients, severity of pain and symptoms, and activities which increase pain and symptoms, are of more use as this potentially reflects the patients’ perceptions of his/her conditions, rather than quantifies pain and symptoms which may not always be present or present at the time of testing. Scores obtained from the SA-PCS, can therefore be related to the severity of the subject’s pain and symptoms, and to the difficulty subjects may experience in performing daily activities. The relationship between catastrophization, avoidance of a particular activity and the influence fibromyalgia has on an individual’s life, may therefore be more natural determined than momentarily-based experiences. The results of this study show that the SA-PCS related with pain severity, fear-avoidance behaviours and impact of fibromyalgia in an expected manner. However, the results for the concurrent validation of the SA-PCS in relation to the SA-TSK and SA-FIQR in this study should therefore be viewed with caution, as the SA-TSK and SA-FIQR were validated at the same time as the SA-PCS.

Another limitation to this study is that the SA-PCS was validated among English, Afrikaans and Xhosa-speaking patients with fibromyalgia living in and around the western parts of South Africa and not the entire South Africa. For this reason, the current validated SA-PCS may not be applicable for patients with fibromyalgia residing in other parts of South Africa due to the vast differences in cultures and languages between various provinces of South Africa. Further cultural and linguistic validation of the SA-PCS for other areas of South Africa is therefore required.

Factor analysis was not performed and may be deemed as a limitation. However, according to DeCoster (1998) the objectives for doing exploratory and confirmatory analysis may not actually apply to this study [58]. Basically, exploratory factor analysis should be used when one is interested in making statements about the factors that are responsible for a set of observed responses. The primary objectives of an exploratory factor analysis are to determine: 1) the number of common factors influencing a set of measures; and 2) the strength of the relationship between each factor and each observed measure. Some common uses of exploratory factor analysis are to: 1) identify the nature of the constructs underlying responses in a specific content area; 2) determine what sets of items \hang together" in a questionnaire; 3) demonstrate the dimensionality of a measurement scale. Researchers often wish to develop scales that respond to a single characteristic; 4) determine what features are most important when classifying a group of items; and 5) generate\factor scores representing values of the underlying constructs for use in other analyses. Since this was not the case in this study, exploratory factor analysis was not conducted. On the other hand, confirmatory factor analysis should be used when one has large numbers of data. The primary objective of a confirmatory factor analysis is to determine the ability of a predetermined factor model to an observed set of data. Some common uses of confirmatory factor analysis are to: 1) establish the validity of a single factor model; 2) compare the ability of two different models to account for the same set of data; 3) test the significance of a specific factor loading; 4) test the relationship between two or more factor loadings; and 5) test whether a set of factors are correlated or uncorrelated [58]. Since items were not added or removed, the factors within the outcome measures essentially remained the same, hence factor analysis was not deemed necessary in this study.

Lastly, there are always queries regarding the appropriate sample size for validation studies, particularly when it is inappropriate to estimate the extent of cultural differences on instrument construction. Therefore, despite the encouraging results of this study, further validation of the SA-PCS should include larger samples per language and ethnic group, and chronic pain conditions other than fibromyalgia.

## Conclusion

The current study findings indicate that the cross-culturally adapted English, Afrikaans and Xhosa SA-PCS showed good face and content validity, excellent internal consistency (for subsections and total PCS), excellent test-retest reliability; as well as satisfactory sensitivity-to-change and cross-sectional convergent validity among patients with fibromyalgia. The SA-PCS can therefore be recommended as simple, efficient, valid and reliable tool which shows satisfactory sensitivity-to-change, for use among English, Afrikaans and Xhosa-speaking patients with fibromyalgia attending the public health sector in the Western Cape area of South Africa. Due to the vast diversity in language, culture and ethnicity evident in South Africa, additional cross-cultural adaptation and validation of the SA-PCS is required for application in the fibromyalgia and chronic pain populations living in other provinces of South Africa. Further testing of the psychometric properties of the English, Afrikaans and Xhosa SA-PCS is warranted to confirm recommendations regarding its use in research.

## Abbreviations

PCS: Pain catastrophizing scale; TSK: Tampa scale for Kinesiophobia; FIQR: Revised Fibromyalgia Impact Questionnaire; SA-PCS: South African Pain catastrophizing scale; SA-TSK: South African Tampa scale for Kinesiophobia; SA-FIQR: South African Revised Fibromyalgia Impact Questionnaire; ICC: Intraclass coefficient correlation; ACR: American College Rheumatology; GPPAQ: General Practice Physical Activity Questionnaire; CI: Confidence Interval; SEM: Standard error of measurement; MDC: Minimum detectable change; SD: Standard deviation; MD: Mean difference; MRC: Medical Research Council; NRF: National Research Foundation.

## Competing interest

The authors declare that there are no conflicts of interest.

## Authors’ contribution

LDM conceptualized and conducted the research, and drafted the manuscript in preparation for publication submission. QAL and KGS assisted in the conceptualization of the research and in drafting the final manuscript. MJS assisted in the logistics of conducting the cross-cultural adaptation of the SA-PCS. All the authors read and approved the final manuscript.
